# Laser Directed Energy Deposition of Inconel625 to Ti6Al4V Heterostructure via Nonlinear Gradient Transition Interlayers

**DOI:** 10.3390/ma18245598

**Published:** 2025-12-12

**Authors:** Wenbo Wang, Guojian Xu, Yaqing Hou, Chenyi Zhang, Guohao Cui, Pengyu Qin, Juncheng Shang, Xiuru Fan

**Affiliations:** 1 School of Mechanical Engineering & Automation, Dalian Polytechnic University, Dalian 116000, China; wangwenbo@dlpu.edu.cn (W.W.); zcystudy166@163.com (C.Z.); cuiguohao01@163.com (G.C.); qinpengyuqas@163.com (P.Q.); sjuncheng@yeah.net (J.S.); 2 School of Materials Science and Engineering, Shenyang University of Technology, Shenyang 110870, China; xuguojian@sut.edu.cn; 3 Research Institute of Advanced Materials (Shenzhen) Co., Ltd., Shenzhen 518045, China; houyaqingwork@163.com

**Keywords:** heterostructure, laser directed energy deposition, gradient transition, phase composition, metallurgical bonding

## Abstract

Heterostructure (HS) refers to a class of structural materials composed of two or more different chemical components or crystal structures. Integration of Inconel 625 (IN625) nickel-based superalloy and Ti6Al4V (TC4) titanium alloy to a HS material offers a promising strategy to achieve graded thermo-mechanical properties, extended service temperature ranges, and significant weight reduction, which are highly desirable in aerospace applications. However, obtaining a better metallurgical bonding between the two alloys remains a critical challenge. In this study, laser directed energy deposition (L-DED) technology was employed to fabricate IN625/TC4 HS materials with a nonlinear gradient transition, following systematic investigations into the phase composition and crack sensitivity of IN625/TC4 gradient layers prepared from mixed powders of varying compositions. In addition, microstructure, phase distribution, and mechanical properties of HS materials at room temperature were characterized. The metallurgical defect-free IN625/TC4 HS material was successfully prepared, featuring a smooth transition of microstructure, reduced cracking sensitivity, and reliable metallurgical bonding. Furthermore, a novel design concept and illustrative reference for the L-DED fabrication of N625/TC4 HS material with excellent comprehensive performance was presented, while providing a theoretical metallurgical basis and data support for the potential applications of IN625/TC4 HS materials in the field of aerospace.

## 1. Introduction

To meet the technical requirements of components in aerospace applications, new functional materials that combine high performance and multifunctionality are under development. Heterostructure (HS) materials consisting of two or more materials attract attention because of the combination of the advantages in mechanical, thermal, and chemical properties [1,2,3,4]. Due to their state-of-the-art performance in high-temperature components and light components, respectively, the integration of nickel-based superalloys and titanium alloys is garnering significant attention as a promising candidate for HS materials [5,6,7,8]. Ni-based superalloys possess significant mechanical properties, creep resistance, fatigue resistance, corrosion resistance, and oxidation resistance, which render them suitable for applications in high-temperature environments, effectively meeting the stringent requirements for advanced engineering components [9,10]. With high specific strength, good corrosion resistance, and thermal stability, titanium alloy has been applied in the medium-temperature and light components [11,12]. Thus, the application of Ni-/Ti-based HS material could effectively improve the thrust-weight ratio (TWR; defined as the ratio of engine thrust to total weight, a key performance metric in aerospace propulsion systems) of aircraft by extending the service temperature range and enhancing the performance [13]. The traditional methods of preparing HS materials include laser welding [14,15], diffusion welding [16], friction welding [17,18], and brazing [19,20], etc. However, such technologies are limited in terms of manufacturing scope, flexibility, and efficiency. Furthermore, IN625 and TC4 alloys exhibit great differences in physical and chemical properties, as well as in their crystal structure, which leads to high residual stresses during the preparation of Ni-/Ti-based HS material. Meanwhile, complex intermetallic compounds form at the interface during alloy preparation [21,22,23]. Crack defects are challenging to mitigate in conventional high-strength alloy manufacturing processes, thereby imposing significant restrictions on the advancement and utilization of nickel- and titanium-based high-strength materials.

As an advanced additive manufacturing technology, laser direct energy deposition (L-DED) fulfills the rigorous demands associated with the fabrication of complex geometries and lightweight components for aerospace applications [24,25,26]. Through layer-by-layer deposition, this method employs a high-energy laser beam as the heat source to achieve near-net shaping with alloy powders as the primary raw material [27,28,29]. By controlling the powder composition and processing parameters, the formation of brittle phases and residual stress might be controlled, and the crack resistance of components could be effectively improved then. Thus, L-DED is recognized as a conceivable manufacturing technology for the fabrication of Ni-/Ti-based HS materials [30,31,32].

Extensive research has been undertaken to explore the manufacture/fabrication of TC4/IN718 HS alloy. Direct deposition of IN718 onto TC4 resulted in the formation of brittle Ti-Ni intermetallic compounds, which led to cracking and spalling. Unfortunately, optimization of the printing parameters yielded minimal results in mitigating crack formation [33]. To avoid the detrimental effects of intermetallic compounds, Onuike et al. implemented a composite intermediate transition layer to separate the two base materials. By incorporating vanadium carbide (VC) and suppressing the precipitation of brittle Ti-Ni compounds, TC4/IN718 HS was successfully fabricated with acceptable bonding strength. Notably, the formation of the orthorhombic Cr_3_C_2_ phase was observed at the same time, which might deteriorate the mechanical performance of the TC4/IN718 HS alloy [33].

The gradient transition method was investigated for fabricating the TC4/IN718 HS materials. Multiple results demonstrate that composition variation in the transition layer leads to an increase in residual stress. For instance, obvious macro-cracking was observed in the 40 vol% TC4 + 60 vol% IN718 transition layers [34]. On the other hand, a suitable composition ratio for the transition layer (e.g., 2:8) might reduce the residual stress [35]. In addition, gradient transition layers formed with three mixture compositions (90 vol% TC4 + 10 vol% IN718, 80 vol% TC4 + 20 vol% IN718, and 70 vol% TC4 + 30 vol% IN718) contributed to a crack-free TC4/IN718 HS coating. This was accompanied by a transformation in microstructure from columnar to equiaxed grains and a maximum Vickers hardness of 1030 HV, in contrast to the typical columnar grain structure in single-material L-DED deposits [36].

In summary, a substantial number of intermetallic compounds might precipitate during the direct manufacture of L-DEDed Ni-/Ti-HS materials, leading to cracking and spalling. Employing a gradient transition approach by controlling the composition of the transition layer could mitigate the significant difference in the thermo-physical properties and suppress the formation of intermetallic compounds [35]. Thus, proper gradient transition interlayers might be the key to effectively fabricating L-DEDed Ni-/Ti-HS components/materials. However, current research on the IN718/TC4 HS cannot be fully applicable to the IN625/TC4 HS preparation, and the research on IN625/TC4 HS has not been sufficiently discussed yet.

In this study, the phase composition and crack tendency of deposited materials with compositions ranging from 90 vol% IN625 + 10 vol% TC4 to 10 vol% IN625 + 90 vol% TC4, in increments of 10 vol% TC4, were analyzed. Crack-free L-DEDed IN625/TC4 HS without cracks and other metallurgical defects was successfully prepared using low-crack-sensitivity gradient transition interlayers (90 vol% IN625 + 10 vol% TC4 and 80 vol% IN625 + 20 vol% TC4). The microstructure, phase composition, and room-temperature mechanical properties of the prepared HS were systematically analyzed. By establishing an optimized preparation process for L-DED-fabricated IN625/TC4 HS components, this study lays a technical foundation for the further application of this material.

## 2. Materials and Methods

### 2.1. Experimental Materials

IN625 nickel-based superalloy plates (200 mm × 100 mm × 20 mm) were used as substrates following sequential polishing, chemical cleaning, and vacuum drying. Both IN625 powder and TC4 powder (45–105 μm) were prepared via the rotating electrode method and subsequently vacuum-dried to remove moisture. The chemical compositions of the IN625 and TC4 powders are presented in Table 1.

### 2.2. The Manufacturing of the IN625/TC4 HS

**Figure 1:**
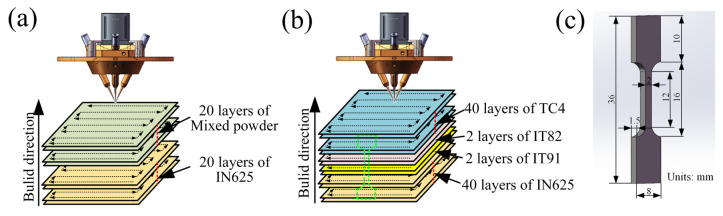
(**a**) Schematic diagram for the preparation of candidate transition layer specimens; (**b**) Schematic diagram of the preparation of IN625/TC4 HS sample; (**c**) Dimensional drawing of the tensile specimen.

The additive manufacturing process of the IN625/TC4 HS was carried out under an inert atmosphere using an LDM-8060 L-DED system (Make: Raycham, Xian, China). The scanning strategy and powder feeding mode employed during laser deposition are illustrated in Figure 1a. To evaluate 9 candidate transition layers with different volume fractions (vol%), specimens were prepared as follows: first, 20 layers of IN625 alloy were deposited on the substrate, followed by the deposition of mixed powder transition layers to form 30 mm × 30 mm × 24 mm specimens with compositions designated in Table 2 (e.g., IT91 to IT19). The proportions of the mixed powders and the corresponding process parameters are presented in Table 2. The process schematic is indicated in Figure 1a. After analyzing the microstructure, phase composition, and cracks of the experimental specimens, two mixed powder proportions with lower crack sensitivity were chosen as the transition layers for the subsequent preparation of the IN625/TC4 HS materials. As shown in the schematic diagram in Figure 1b, 40 layers of IN625 alloy were first deposited onto the substrate. Subsequently, 2 layers of each selected transition interlayer material were deposited, followed by the deposition of 40 layers of TC4 alloy to produce 40 mm × 40 mm × 50 mm specimens.

### 2.3. Characterization Methods

Metallographic specimens were cut, ground with sandpapers (400#–5000#), and polished with SiO_2_ suspension. They are subsequently subjected to a two-step process: first with Kroll reagent for 15–20 s, followed by aqua regia for 30–35 s. Optical microscopy was conducted after both etching steps to reveal the overall microstructure. A ZX-10 Zeiss optical microscope (OM) (Make: Zeiss, Jena, Germany) and an SU8010 (Make: Hitachi, Naka, Japan) field emission scanning electron microscope (SEM) were used for microstructure observation, chemical composition analysis, and fracture morphology analysis. Phase composition analysis was conducted utilizing an XRD-7000 X-ray diffractometer (XRD) (Make: Shimadzu, Kyoto, Japan) with the following parameters: scanning range of 30–90°; scanning speed of 4°/s; radiation source of Cu-Kα (λ = 1.5406 Å), and focal spot size of 1.0 × 10 mm^2^. For phase composition and distribution analysis, an Ilion II 697 argon ion beam cross-section polisher (Make: Gatan, Pittsburgh, Pennsylvania, USA) was used to prepare a damage-free surface, followed by Electron Backscattered Diffraction (EBSD) analysis. An AZtecCrystal software (Version: 2.1, Make: Oxford Instruments plc, Abingdon, Oxfordshire, UK) was used to process and analyze the EBSD data. Tensile specimens were cut along the Building Direction (BD) via wire electrical discharge machining. The sampling positions and method are shown in Figure 1b and Figure 1c, respectively. After grinding and polishing, tensile tests were conducted using a WDW-100 microcomputer-controlled tensile testing machine (Make: Make: Kexin, Changchun, China) at a crosshead speed of 0.1 mm/min. At least three specimens were evaluated for accuracy, and the average tensile strength was calculated. The hardness of the specimens was tested using an HVS-1000B Vickers hardness tester (Make: Zhijin, Laizhou, China) with a load of 10 kg and a dwell time of 10 s. Five points were measured in each horizontal direction, and the average values were then calculated.

## 3. Results and Discussion

### 3.1. Selection of Transition Layers

**Figure 2:**
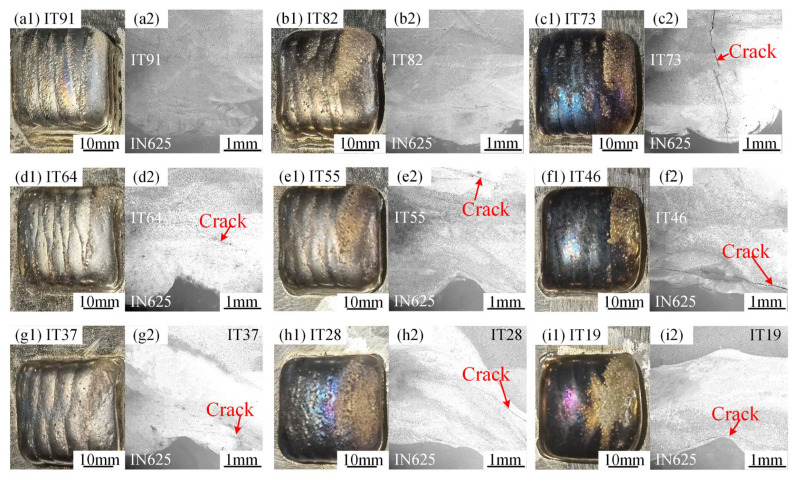
Photograph and the low-magnification SEM images of the candidate transition layers: (**a1**) Sample surfaces of IN625/IT91. (**a2**) Cross-sections of IN625/IT91. (**b1**) Sample surfaces of IN625/IT82. (**b2**) Cross-sections of IN625/IT82. (**c1**) Sample surfaces of IN625/IT73. (**c2**) Cross-sections of IN625/IT73. (**d1**) Sample surfaces of IN625/IT64. (**d2**) Cross-sections of IN625/IT64. (**e1**) Sample surfaces of IN625/IT55. (**e2**) Cross-sections of IN625/IT55. (**f1**) Sample surfaces of IN625/IT46. (**f2**) Cross-sections of IN625/IT46. (**g1**) Sample surfaces of IN625/IT37. (**g2**) Cross-sections of IN625/IT37. (**h1**) Sample surfaces of IN625/IT28. (**h2**) Cross-sections of IN625/IT28. (**i1**) Sample surfaces of IN625/IT19. (**i2**) Cross-sections of IN625/IT19.

Figure 2(a1–i1) display macroscopic views of the nine candidate transition layer specimens with varying composition ratios, while Figure 2(a2–i2) show the corresponding SEM images captured along the deposition direction. No cracks were found in the structure from the side of IN625 to the side of the mixed powder deposition layer in IT91 (90 vol% IN625 + 10 vol% TC4) and IT82 (80 vol% IN625 + 20 vol% TC4). When the TC4 content increased to 30 vol% (IT73: 70 vol% IN625 + 30 vol% TC4), longitudinal cracks were observed through the sedimentary layer, and the transverse cracks were observed in the range of 40–50 vol% TC4 specimens. With further increases in the TC4 content to 60–90 vol%, multiple transverse cracks were observed in the transition region adjacent to the IN625 matrix. The tests above were repeated at least five times, and the test results—especially crack formation in each group—exhibited good consistency.

**Figure 3:**
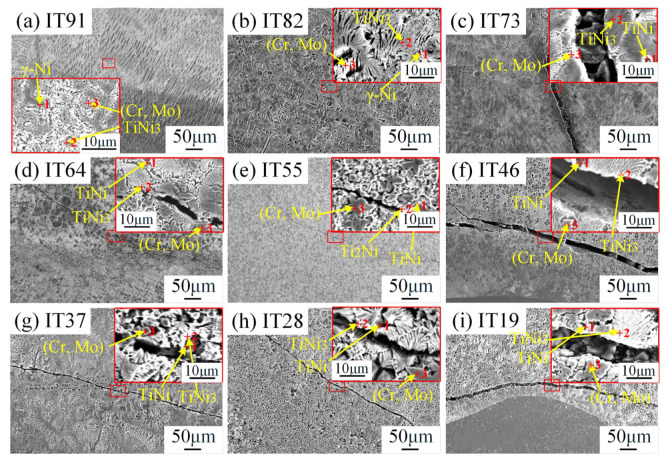
SEM image of transition layers: (**a**) IT91; (**b**) IT82; (**c**) IT73; (**d**) IT64; (**e**) IT55; (**f**) IT46; (**g**) IT37; (**h**) IT28; (**i**) IT19.

Microstructure and element distribution of the specific position were analyzed and displayed in Figure 3 and Table 3. The deposits with 90 vol% IN625 (10 vol% TC4) and 80 vol% IN625 (20 vol% TC4) are composed of γ-Ni dendrites, TiNi3, and (Cr, Mo) precipitates, respectively. Among these, the Ti-Ni brittle phases and γ-Ni in the 80 vol% IN625 deposited layer formed a eutectic structure. In the deposition layer of 70 vol% IN625 (c.f. Figure 3c), the region adjacent to the crack consists of a (TiNi_3_ + TiNi) eutectic structure and (Cr, Mo) precipitated phases along the grain boundaries. The longitudinal crack in this area gradually extends downward from the top, passes through the deposition layer, and terminates above the IN625 region. The phase composition adjacent to the crack of the deposition layer of 60 vol%IN625 was consistent with that in the 70 vol% IN625 specimen. Further increasing the TC4 ratio to 50 vol%, TiNi, Ti_2_Ni, and (Cr, Mo) phases are observed. In addition, deposition layers with more than 60 vol% of TC4 are primarily composed of (TiNi + TiNi_3_) eutectic and a small amount of massive (Cr, Mo) phases. The (TiNi + TiNi_3_) eutectic structure exhibits a lamellar morphology. The alloy composition and microstructure morphology indicate that the eutectic structure belongs to the typical metal-metal type two-phase eutectic structure. In the formation process of such an eutectic structure, the liquid-solid interface shows a rough–rough interface, and the eutectic two phases alternately nucleate during the solidification process; and the growth process is to multiply the lamellae of the same phase by the bridging method. At the same time, due to the rapid melting and solidification characteristics of L-DED technology, the deposition layer behaves undercooling, resulting in the formation of a fine lamellar eutectic structure. As displayed in Figure 3, Cr and Mo are enriched in the bulk phase, while Ti and Ni are mainly distributed in the eutectic structure. Such segregation arises from insufficient elemental diffusion during rapid solidification, which reduces the crack resistance of the material and leads to cracking.

**Figure 4:**
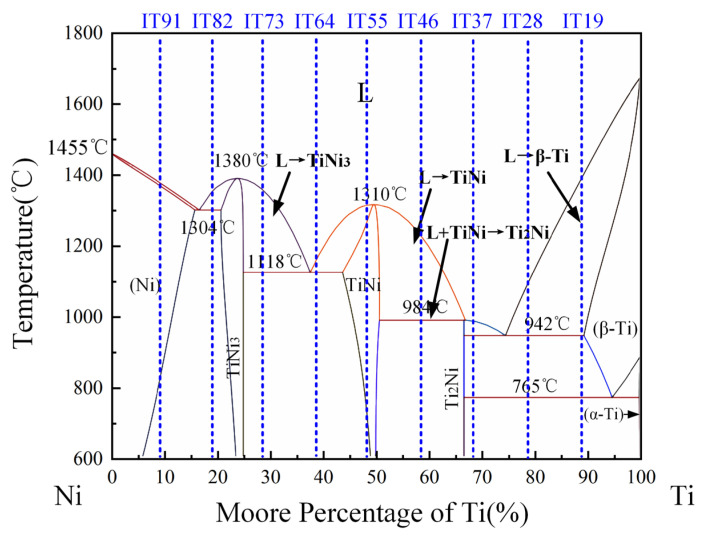
Phase diagram of Ti-Ni binary alloy.

The Ti-Ni binary phase diagram was calculated by commercial thermodynamic modeling software Thermocalc^®^2021a (Thermo-Calc Software AB, Stockholm, Sweden). Chemically varying alloys were thermodynamically calculated based on the CALPHAD method utilizing the TCS Steel Database TCFE10, and equilibria and stability of phases were calculated [38,39]. Referring to the phase diagram (cf. Figure 4), IN625/TC4 presents a complex alloy system: (1) β-Ti → α-Ti + Ti_2_Ni eutectoid transition at 765 °C; (2) L → β-Ti + Ti_2_Ni and L → TiNi + TiNi_3_ eutectic transitions at 942 °C and 1118 °C, respectively; (3) L + TiNi → Ti_2_Ni pericardial transition occurs at 984 °C, and (4) L → TiNi_3_ + γ-Ni eutectic transition occurs at 1304 °C. The brittle intermetallic compounds such as Ti_2_Ni, TiNi, and TiNi_3_ formed by Ti and Ni severely affect the properties of materials. At low TC4 proportion (10–20%), the microstructure of such transition layers mainly contains γ-Ni, TiNi_3_, and (Cr, Mo) phase; no cracks were observed, which is consistent with the prediction of the Ti-Ni phase diagram. When the ratio of TC4 increases to 30–40%, the microstructure is mainly composed of (TiNi + TiNi_3_) eutectic and (Cr, Mo) phase, where cracks started to appear. In IT55, consistent with the phase diagram, the TiNi + Ti_2_Ni phase was observed near the crack. The internal stress increases along with the increase in the brittle phase, which leads to an increase in crack sensitivity. When the ratio of TC4 increased to 60–90%, high laser power was applied, which led to an increase in the dilution rate of IN625 and the formation of (TiNi + TiNi_3_) eutectic and (Cr, Mo) phase in the transition region. At the same time, the increased difference in thermal expansion coefficients caused stress concentration, leading to transgranular cracks.

In summary, crack formation in the gradient structure is mainly attributed to the formation of the brittle phases and the accumulation of internal stress. However, the L-DED process inevitably introduces internal stress, which makes reducing the brittle phase the key to crack inhibition. Therefore, IT91 (90 vol% IN625 + 10 vol% TC4) and IT82 (80 vol% IN625 + 20 vol% TC4) with low crack sensitivity were selected as gradient transition interlayers to fabricate IN625/TC4 HS materials.

### 3.2. Microstructure Analysis

**Figure 5:**
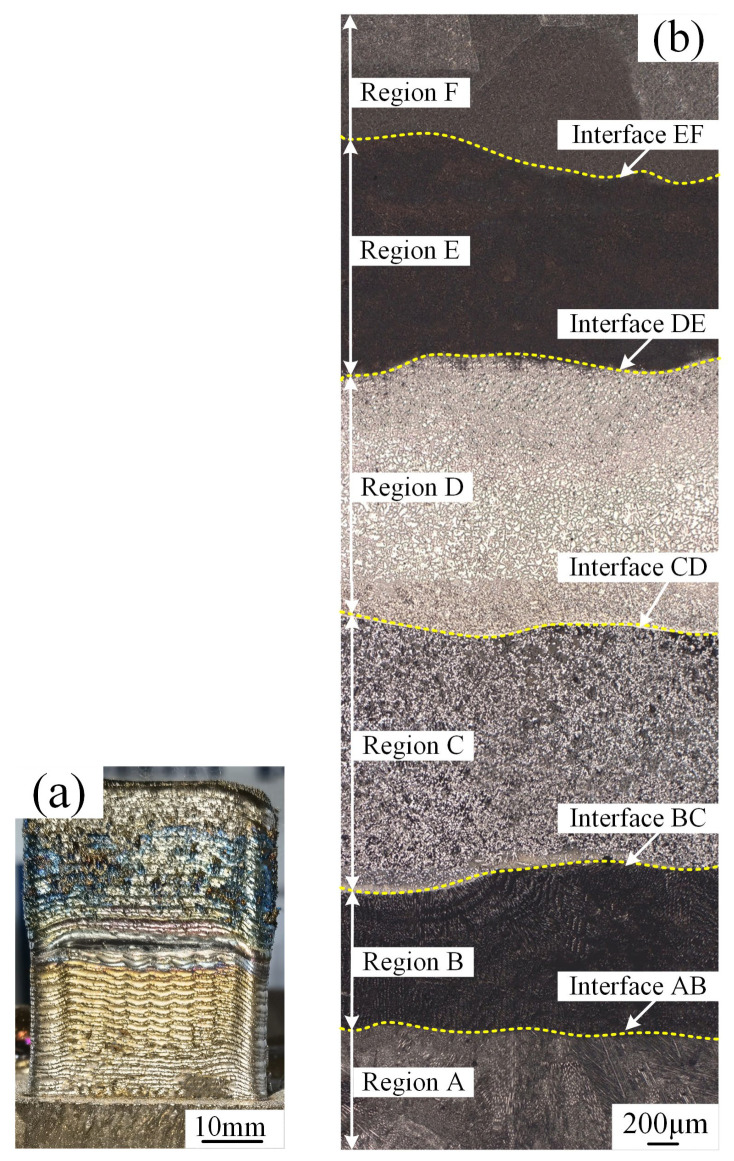
Image of IN625/TC4 HS specimen: (**a**) macroscopic photograph; (**b**) optical micrograph microstructure.

The macroscopic image and optical micrograph image of the IN625/TC4 HS specimen are displayed in Figure 5a and 5b, respectively. Along the deposition direction, morphological changes could be observed. Depending on the process parameter and structure characteristics, the HS specimen is divided into six regions along the deposition direction and five interfaces in between (c.f. Figure 5b): Region A was pure IN625; Region B and Region C were the IT91 and IT 82 regions, respectively; Region D and Region E were the lower and upper TC4 gradient transition regions; and Region F was pure TC4.

**Figure 6:**
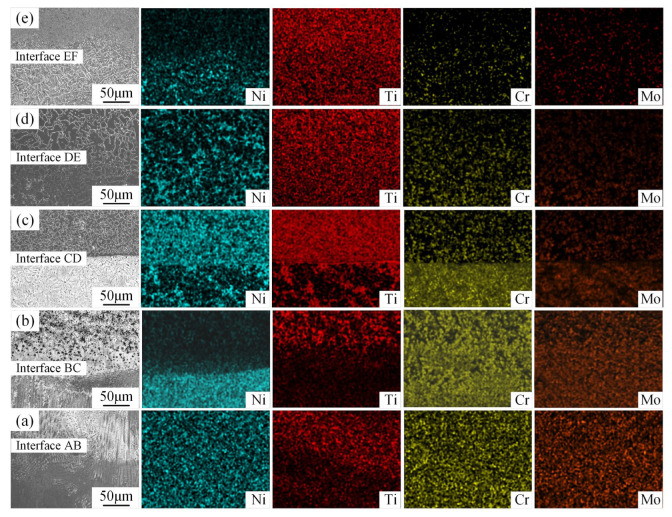
Microstructures and EDS map scanning results near (**a**) interface AB; (**b**) interface BC; (**c**) interface CD; (**d**) interface DE; (**e**) interface EF.

The distribution of elements near each interface was analyzed by EDS scanning (Figure 6). At the Interface AB (Figure 6a), a dendritic structure was observed in the Region A side. While the dendrite structure decreases in the Region B side and the eutectic structure appears, along with the increase in Ti content, Ni, Cr, and Mo content were found to slightly decrease. The Region C above Interface BC (Figure 6b) was dominated by eutectic structure and Cr- and Mo-rich phases, with a decrease in Ni, an increase in Ti, and a marked segregation of Cr and Mo. The microstructure of the D region above the Interface CD (Figure 6c) presented a light gray matrix and dark gray block phase, with a decreasing Cr and Mo content and an increasing Ti content, and a uniform Ni distribution. At the Interface DE (Figure 6d), the upper part represents Region E, which behaved near-equiaxed, with the dispersed distribution of Ti, where the Ni was segregated along the grain boundary. Above the Interface EF (Figure 6e), in the F-region, Ti further increases, and Ni, Cr, and Mo were barely detectable. These observation results showed that the microstructure of each region underwent continuous transformation, and the interfaces were metallurgically bonded.

**Figure 7:**
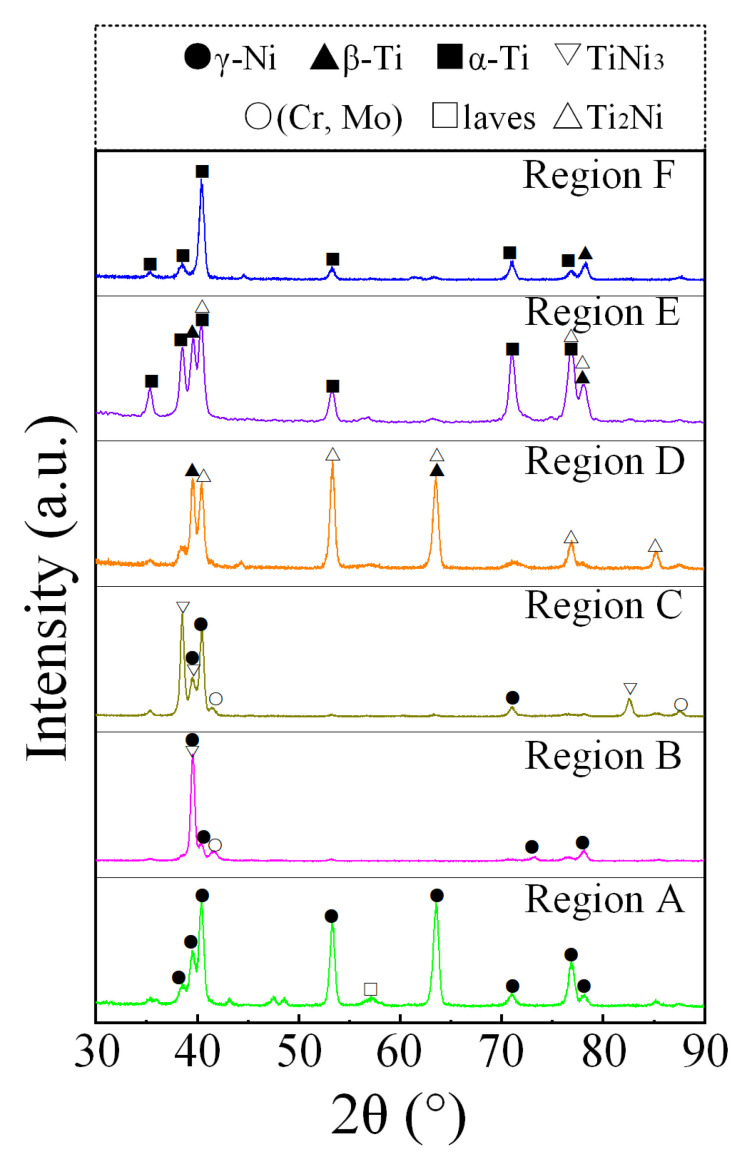
XRD patterns of LMD IN625/TC4 HS with gradient proportions.

XRD analysis (c.f. Figure 7) showed that from Region A to Region F, the microstructure of the HS specimen consisted of the following evolutionary sequence: γ-Ni + Laves → γ-Ni + TiNi_3_ + (Cr, Mo) → TiNi_3_ + (Cr, Mo) + γ-Ni → β-Ti + Ti_2_Ni → β-Ti + Ti_2_Ni + α-Ti → α-Ti + β-Ti. Among these regions, Region A and Region F were consistent with the characteristics of IN625 and TC4 alloy, respectively. The phase compositions of Region B and Region C were consistent with the Ti-Ni binary phase diagram. In Region D and Region E, the Ti_2_Ni intermetallic compound appeared.

**Figure 8:**
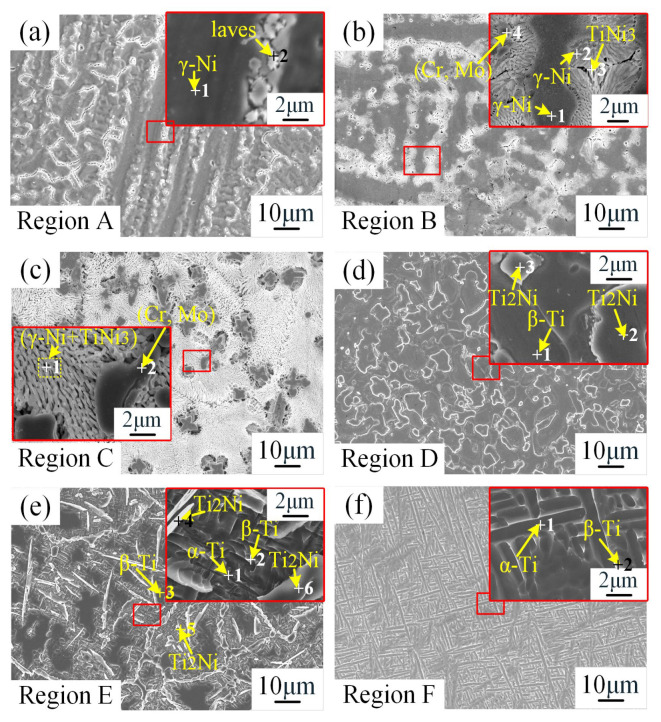
Microstructure and enlarged view of (**a**) region A; (**b**) region B; (**c**) region C; (**d**) region D; (**e**) region E; (**f**) region F.

To conduct a detailed investigation of the microstructure and phase distribution in each region of the TC4/IN625 gradient HS specimen, high-magnification scanning electron microscopy observation (c.f. Figure 8) and EDS point scanning analysis (c.f. Table 4) were performed. The microstructure and phase composition of each region were inferred as follows: Region A was mainly composed of gray γ-Ni dendrites (Point a1: high Ni, Cr content) and interdendritic Laves phases (Point a2: Mo/Nb/Fe/Ti-rich); Region B contained dark gray γ-Ni columnar dendrites (Point B1), (γ-Ni + TiNi_3_) eutectic (TiNi_3_ at Points B2 and B3: Ni/Ti ≈ 3:1), and a small amount of (Cr, Mo)-rich phases (Point B4: high Cr, Mo content); The main phase of Region C was (γ-Ni + TiNi_3_) eutectic (Region C1), with a small amount of blocky (Cr, Mo)-rich phases (Point C2: higher Cr, Mo content than in Region B); Region D was mainly composed of a light gray β-Ti matrix (Point D1: high Ti content) and Ti_2_Ni precipitates (Point D2: Ti/Ni ≈ 2:1); Region E consisted of α-Ti (Point E1: high Ti, Al content), β-Ti (Points E2 and E3: high Ti content and β-stabilizing elements), and Ti_2_Ni (Points E4–E6: Ti/Ni ≈ 2:1); Region F consisted of α-Ti (Point f1: Al-rich) and β-Ti (Point f2: V-rich) phases.

**Figure 9:**
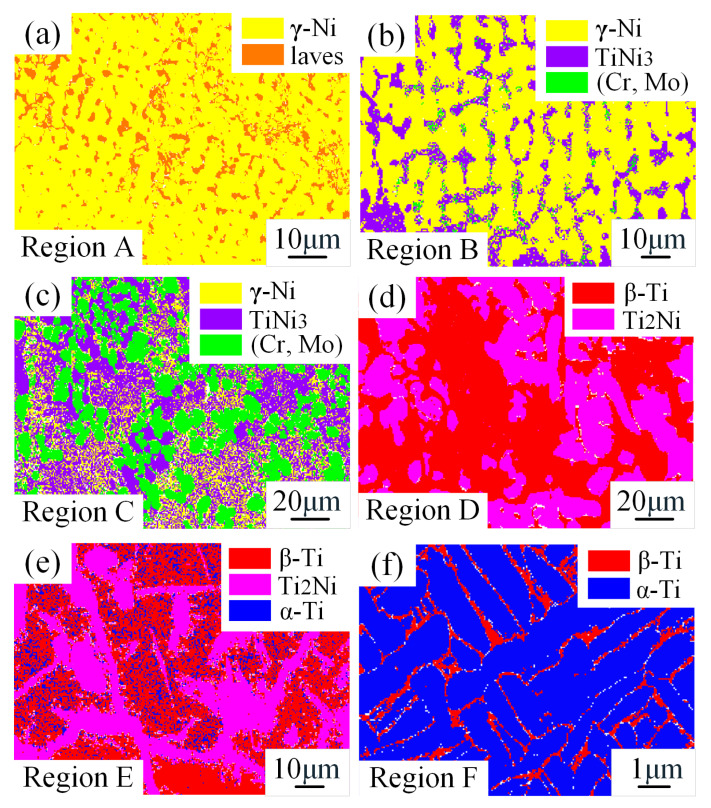
EBSD phase analysis results of (**a**) Region A; (**b**) Region B; (**c**) Region C; (**d**) Region D; (**e**) Region E; (**f**) Region F.

BSD analysis (Figure 9 and Table 5) was performed to further clarify the phase distribution and quantitative composition of each region. The results indicated that Region A (Figure 9a) was mainly composed of γ-Ni dendrites (81.4%) and a small amount of Laves phase (17.1%); Region B (Figure 9b): γ-Ni existed as columnar dendrites (73.2%), with interdendritic (γ-Ni + TiNi_3_) eutectic (TiNi_3_ ~23.0%) and minor (Cr, Mo)-rich phases (3.0%) observed. With the addition of 10 vol% TC4, the dendrites in this region transformed into columnar dendrites, and the eutectic reaction L → γ-Ni + TiNi_3_ occurred. Ti and Ni combined to form TiNi_3_, which induced Cr and Mo segregation, leading to the formation of fine (Cr, Mo)-rich phases; Region C (Figure 9c) was dominated by massive (Cr, Mo)-rich phases (38.4%) and (γ-Ni + TiNi_3_) eutectic, with γ-Ni and TiNi_3_ accounting for 19.2% and 38.1%, respectively. As the TC4 content increased to 20 vol%, eutectic structures formed, and the enrichment of Cr and Mo increased, accompanied by phase coarsening; Region D (Figure 9d) mainly consisted of β-Ti (57.7%) and Ti_2_Ni (39.4%). Due to the higher laser power and dilution rate in this region, elements diffused upward to form β-Ti and Ti_2_Ni, with the corresponding eutectic reaction: L → β-Ti + Ti_2_Ni; Region E (Figure 9e) was composed of β-Ti (51.3%), Ti_2_Ni (35.1%), and a small amount of α-Ti (9.3%). With the increase in TC4 layer thickness, the upward diffusion of alloying elements decreased, and the contents of β-Ti and Ti_2_Ni were lower than those in Region D; Region F (Figure 9f) exhibited typical microstructural features of TC4, including α-Ti (90.6%) and a small amount of β-Ti (8.7%).

In summary, by systematically characterizing the IN625/TC4 gradient heterostructures (HS) material prepared utilizing laser-directed energy deposition (L-DED), this research demonstrated that introducing gradient transition interlayers can suppress the formation of the metallic compound and metallic compound eutectic structure in the transition zone. Although the formation of intermetallic compounds (e.g., TiNi_3_, Ti_2_Ni, and the (Cr, Mo) phase) is barely avoidable due to the inherent thermodynamic interaction between the two alloy systems and the intrinsic characteristics of the L-DED process, the type and quantity of intermetallic compounds could be effectively reduced via the optimization of interlayer composition. In addition, the gradient transition layer could alleviate discrepancies in the thermal expansion coefficient between IN625 and TC4 alloys, thereby reducing the internal stress of the transition zone. Thus, the crack sensitivity of HS materials decreased, and a reliable metallurgical bonding of the dissimilar HS was achieved.

### 3.3. Mechanical Properties

**Figure 10:**
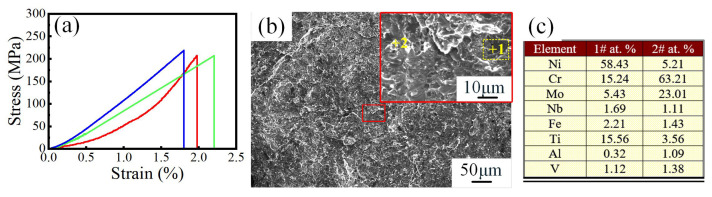
Tensile test investigation: (**a**) stress–strain curve; (**b**) the morphology of the tensile fracture surface; (**c**) EDS results of the two points in (**b**).

The tensile properties of gradient transition IN625/TC4 HS materials were investigated. As shown in the stress–strain curve of the tensile specimen (c.f. Figure 10a), no obvious yield stage was observed [40], and the average tensile strength of the specimen reached approximately 211 MPa. Fracture analysis revealed that cleavage steps and tearing edges were observed on the fracture (c.f. Figure 10b), which indicates a quasi-cleavage fracture. Figure 10c presents the EDS composition analysis of the two different microdomains (as shown in Figure 10c). Combined with previous analyses, Point 1# could be identified as a eutectic structure of γ-Ni and TiNi_3_, and Point 2# could be identified as a (Cr, Mo) phase. The fracture surface exhibited similarities in structure and atomic ratio of Cr and Mo to those in region C, which indicates the fracture was likely located in region C of the HS specimen. During laser processing, significant residual stress was generated due to the difference in thermal expansion coefficients between dissimilar metals and the phase transformation-induced stress. The brittle intermetallic compounds formed in the transition zone were suspected to be the crack initiation sources under the combined action of residual stress and external loads during the tensile test. Meanwhile, the low-melting-point eutectic formed by γ-Ni and TiNi_3_ also promotes the formation of cracks.

**Figure 11:**
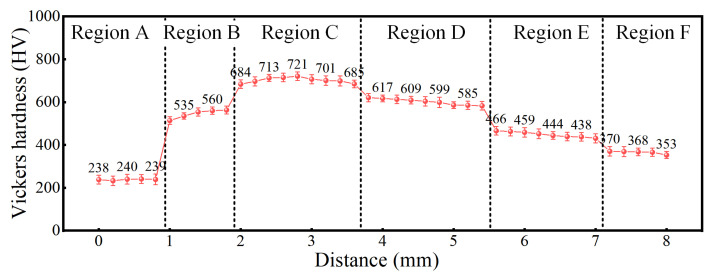
Vickers hardness distribution of IN625/TC4 HS with gradient proportions.

For further investigation of the impact of microstructure on the mechanical properties of HS materials, the Vickers hardness profile along the BD for the gradient transition L-DEDed IN625/TC4 HS is displayed in Figure 11. The Vickers hardness test was conducted starting at the bottom of the structure and moving upward along the BD at 0.2 mm intervals. The Vickers hardness distribution across the six regions (A → F) of the gradient transition L-DEDed IN625/TC4 HS exhibited a non-monotonic trend, initially increasing and then decreasing. The highest hardness is observed in region C, with a peak value of approximately 721 HV and an average value of about 702 HV. Region D exhibited the second-highest average hardness of approximately 602 HV. In contrast, region A exhibited the lowest average hardness of approximately 238 HV, while region F ranks as the second softest with an average value of 365 HV. Regions B and E demonstrate intermediate hardness levels, with average values of approximately 545 HV and 449 HV, respectively, both of which exceeded those of the monolithic IN625 and TC4 base alloys [41,42].

After detailed analysis of the hardness in each region, the mechanical properties of the IN625/TC4 HS specimen exhibited a corresponding relationship to the phase composition of each region. Region A is mainly composed of γ-Ni solid solutions with an FCC structure and a small amount of Laves phase. The Vickers hardness value of this region is consistent with the hardness characteristics of IN625 alloy prepared by L-DED. Introducing the TC4 alloy, Region B and Region C contain the TiNi_3_ compound and the (Cr, Mo)-phase in the γ-Ni solid solution matrix. The intermetallic phases normally exhibit higher hardness but lower ductility and contribute to dispersion strengthening. Thus, due to the increase in the quantity and size of intermetallic compounds, the hardness of Region C reaches its peak. For Region D, the dilution rate of the (80% IN625 + 20% TC4) gradient deposition layer increases due to the high laser output power, which leads to the formation of the β-Ti phase and Ti_2_Ni compound. At the same time, the amount of brittle intermetallic phases, such as TiNi_3_ compound and (Cr, Mo)-phase, decreases. For this reason, the hardness starts decreasing. With the continuously increasing TC4 alloy deposition layer, the α-Ti phase starts to form at Region E. Combining with the large amount of β-Ti phase, the hardness in this region keeps decreasing. Finally, the Vickers hardness maintains a consistent value with the hardness characteristics of TC4 alloy prepared by L-DED in Region F, which is composed of a large amount of α-Ti phase and a small amount of β-Ti phase.

Overall, the formation of intermetallic compounds significantly impacts the mechanical properties of HS. The brittle intermetallic compounds are the main source of crack initiation and propagation; thus, this directly determines the strength, hardness, and fracture behavior of HS. The distribution of intermetallic compounds is influenced by manufacturing processes and elemental interactions. Therefore, optimizing the manufacturing process and adding intermediate layers could regulate the microstructure of the HS alloys and further achieve the desired mechanical properties.

## 4. Conclusions

In this study, the phase composition and crack sensitivity of IN625/TC4 mixed powders with volume increments of 10% TC4 in the laser directional energy deposition (L-DED) process were systematically investigated. Based on the crack evaluation results, the low crack sensitivity components (90% IN625 + 10% TC4 and 80% IN625 + 20% TC4) were selected to serve as the nonlinear gradient transition interlayers, and the IN625/TC4 gradient heterostructure (HS) free of metallurgical defects was successfully prepared. The microstructure, phase distribution, and mechanical properties of HS materials at ambient temperature were analyzed. The main conclusions are as follows:

(1) In the deposition layer of 90% IN625 + 10% TC4 and 80% IN625 + 20% TC4, the structure presents no cracks; mainly γ-Ni, TiNi_3_, and (Cr, Mo) phases were observed. With the increase in TC4 content (30–90%), a large number of Ti-Ni intermetallic compounds and (Cr, Mo) brittle phases formed in the material, which leads to transgranular cold cracks and a significant increase in crack sensitivity.

(2) With proper translation layers, IN625/TC4 HS alloys without cracks were prepared. The specimen can be divided into six continuous regions from the IN625 side to the TC4 side as γ-Ni + Laves → γ-Ni + TiNi_3_ + (Cr, Mo) → TiNi_3_ + (Cr, Mo) + γ-Ni → β-Ti + Ti_2_Ni → β-Ti + Ti_2_Ni + α-Ti → α-Ti + β-Ti.

(3) The precipitation of Ti-Ni intermetallic compounds and the (Cr, Mo) phase in the transition region leads to a significantly higher hardness than that of the substrate on both sides, with a peak value of 721 HV. The tensile strength of the HS specimen at room temperature is about 211 MPa, and the fracture morphology is quasi-cleavage fracture.

(4) The nonlinear gradient transition contributes to the smooth transition of microstructure, the decrease in cracking sensitivity, and the accomplishment of metallurgical bonding of differential HS preparation. Optimization of process parameters, composition design, and heat treatment would help to further depress the undesired intermetallic compounds and improve the mechanical properties of HS materials.

(5) The research results of this paper can provide new design ideas for the preparation of IN625/TC4 HS components and offer data support for future applications.

## Figures and Tables

**Table 1:** Chemical compositions of IN625 and TC4 alloy powders (wt. %) [37].

	Mass Fraction (%)								
IN625	C	Fe	Al	Mo	Nb	Cr	Si	Ti	Ni
	0.05	4.01	0.30	8.85	3.59	22.1	0.02	0.21	Bal.
TC4	C	Fe	Al	O	H	N	V	Ti	
	0.007	0.206	6.26	0.082	0.004	0.006	4.08	Bal.	

**Table 2:** Processing parameters for experimental composition.

Name	Material Composition (vol.%)	Laser Power (W)	Scan Speed (mm/min)	Layer Thickness (mm)	Protective Gas Flow Rate (L/min)
IN625	100%IN625	1800	720	0.6	15
IT91	90%IN625 + 10%TC4	1850	720	0.6	15
IT82	80%IN625 + 20%TC4	1900	720	0.6	15
IT73	70%IN625 + 30%TC4	1950	720	0.6	15
IT64	60%IN625 + 40%TC4	2000	720	0.6	15
IT55	50%IN625 + 50%TC4	2050	720	0.6	15
IT46	40%IN625 + 60%TC4	2100	720	0.6	15
IT37	30%IN625 + 70%TC4	2150	720	0.6	15
IT28	20%IN625 + 80%TC4	2200	720	0.6	15
IT19	10%IN625 + 90%TC4	2250	720	0.6	15
TC4	100%TC4	2300	720	0.6	15

**Table 3:** EDS component analysis results of the microstructure of the 9 candidate transition layer specimens (at. %).

Location		Ni	Cr	Mo	Nb	Fe	Ti	Al	V	Deduced Phases
IT91	a1	59.78 ± 1.54	20.43 ± 1.03	6.56 ± 0.53	2.14 ± 0.04	3.21 ± 0.07	3.21 ± 0.09	2.43 ± 0.04	2.24 ± 0.03	γ-Ni
	a2	62.89 ± 2.36	6.78 ± 0.66	4.32 ± 0.12	3.21 ± 0.07	1.01 ± 0.02	18.21 ± 0.98	2.01 ± 0.03	1.57 ± 0.06	TiNi_3_
	a3	8.91 ± 0.78	67.01 ± 2.54	16.25 ± 0.99	1.01 ± 0.04	1.13 ± 0.02	2.46 ± 0.06	1.59 ± 0.04	1.64 ± 0.03	(Cr, Mo)
IT82	b1	55.21 ± 1.87	20.31 ± 1.01	8.34 ± 0.87	3.21 ± 0.09	4.11 ± 0.18	5.96 ± 0.59	1.53 ± 0.06	1.33 ± 0.02	γ-Ni
	b2	60.32 ± 2.56	7.01 ± 0.19	5.59 ± 0.13	2.21 ± 0.09	2.59 ± 0.08	19.32 ± 1.01	1.54 ± 0.02	1.42 ± 0.06	TiNi_3_
	b3	10.21 ± 0.33	64.32 ± 2.98	18.53 ± 0.98	1.01 ± 0.02	2.31 ± 0.06	1.21 ± 0.03	1.03 ± 0.02	1.38 ± 0.02	(Cr, Mo)
IT73	c1	50.71 ± 1.98	1.59 ± 0.09	1.06 ± 0.06	1.54 ± 0.05	1.32 ± 0.05	41.21 ± 1.87	1.55 ± 0.02	1.02 ± 0.03	TiNi
	c2	60.21 ± 2.33	6.64 ± 0.23	3.21 ± 0.15	1.09 ± 0.05	1.11 ± 0.06	23.48 ± 1.01	1.05 ± 0.03	3.21 ± 0.04	TiNi_3_
	c3	4.22 ± 0.07	65.32 ± 2.68	18.43 ± 1.03	1.61 ± 0.09	1.59 ± 0.02	5.03 ± 0.04	2.21 ± 0.04	1.59 ± 0.03	(Cr, Mo)
IT64	d1	49.32 ± 2.01	2.54 ± 0.08	1.11 ± 0.08	1.32 ± 0.06	1.11 ± 0.03	42.21 ± 1.87	1.11 ± 0.06	1.28 ± 0.09	TiNi
	d2	58.24 ± 1.68	9.21 ± 0.25	4.54 ± 0.16	2.25 ± 0.13	2.21 ± 0.16	20.34 ± 1.23	1.54 ± 0.09	1.67 ± 0.07	TiNi_3_
	d3	9.24 ± 0.19	60.24 ± 1.94	20.21 ± 1.01	5.32 ± 0.13	1.59 ± 0.12	1.21 ± 0.14	1.01 ± 0.09	1.18 ± 0.04	(Cr, Mo)
IT55	e1	50.34 ± 1.88	3.24 ± 0.09	1.59 ± 0.07	1.04 ± 0.01	1.05 ± 0.02	40.21 ± 1.84	1.08 ± 0.04	1.45 ± 0.03	TiNi
	e2	31.81 ± 1.89	2.54 ± 0.05	2.02 ± 0.06	1.13 ± 0.03	1.14 ± 0.02	55.53 ± 2.02	3.59 ± 0.08	2.24 ± 0.09	Ti_2_Ni
	e3	10.24 ± 0.51	58.43 ± 1.91	20.34 ± 1.01	3.29 ± 0.09	3.21 ± 0.08	2.22 ± 0.08	1.11 ± 0.02	1.16 ± 0.02	(Cr, Mo)
IT46	f1	45.02 ± 2.31	4.63 ± 0.24	2.50 ± 0.06	3.25 ± 0.07	2.51 ± 0.07	38.35 ± 1.93	2.18 ± 0.04	1.56 ± 0.02	TiNi
	f2	54.54 ± 2.01	6.94 ± 0.07	5.62 ± 0.06	2.62 ± 0.03	1.50 ± 0.05	22.08 ± 0.99	4.33 ± 0.02	2.37 ± 0.04	TiNi_3_
	f3	10.59 ± 0.19	60.23 ± 1.88	18.84 ± 0.97	3.57 ± 0.06	3.21 ± 0.06	1.04 ± 0.03	1.32 ± 0.02	1.20 ± 0.03	(Cr, Mo)
IT37	g1	41.59 ± 1.01	5.56 ± 0.08	3.21 ± 0.03	1.77 ± 0.05	1.94 ± 0.06	41.24 ± 0.87	3.21 ± 0.04	1.48 ± 0.02	TiNi
	g2	58.24 ± 1.69	3.33 ± 0.03	3.21 ± 0.02	2.77 ± 0.06	1.79 ± 0.02	23.24 ± 0.99	4.55 ± 0.12	2.87 ± 0.08	TiNi_3_
	g3	13.34 ± 0.78	55.55 ± 2.43	17.24 ± 0.99	6.64 ± 0.16	2.89 ± 0.32	1.33 ± 0.04	1.34 ± 0.04	1.67 ± 0.05	(Cr, Mo)
IT28	h1	35.43 ± 0.53	6.64 ± 0.16	2.97 ± 0.09	1.94 ± 0.09	1.58 ± 0.08	42.99 ± 1.08	3.54 ± 0.15	4.91 ± 0.18	TiNi
	h2	60.33 ± 2.63	5.59 ± 0.11	1.67 ± 0.08	1.04 ± 0.04	1.99 ± 0.03	21.53 ± 0.89	5.64 ± 0.16	2.21 ± 0.06	TiNi_3_
	h3	10.94 ± 0.25	59.64 ± 2.08	17.31 ± 0.76	5.54 ± 0.26	1.88 ± 0.06	1.69 ± 0.06	1.37 ± 0.05	1.63 ± 0.06	(Cr, Mo)
IT19	i1	36.99 ± 1.36	7.32 ± 0.15	3.84 ± 0.11	2.59 ± 0.14	1.55 ± 0.07	44.68 ± 1.39	1.55 ± 0.05	1.48 ± 0.04	TiNi
	i2	58.34 ± 1.78	6.03 ± 0.14	2.55 ± 0.02	1.87 ± 0.02	1.43 ± 0.03	22.95 ± 0.77	3.24 ± 0.04	3.59 ± 0.09	TiNi_3_
	i3	9.94 ± 0.26	60.33 ± 2.33	17.84 ± 0.87	5.44 ± 0.15	1.21 ± 0.04	2.04 ± 0.06	1.33 ± 0.07	1.87 ± 0.09	(Cr, Mo)

**Table 4:** EDS component analysis results of the microstructure of IN625/TC4 HS (at. %).

Location		Ni	Cr	Mo	Nb	Fe	Ti	Al	V	Deduced Phases
Region A	a1	66.57 ± 2.23	18.09 ± 1.01	2.89 ± 0.09	1.17 ± 0.02	3.75 ± 0.15	4.53 ± 0.13	1.68 ± 0.06	1.32 ± 0.03	γ-Ni
	a2	39.87 ± 1.03	17.24 ± 0.87	14.55 ± 0.19	12.97 ± 0.16	5.77 ± 0.15	7.32 ± 0.16	1.03 ± 0.06	1.25 ± 0.05	laves
Region B	b1	59.52 ± 2.34	23.06 ± 1.01	4.87 ± 0.09	2.68 ± 0.04	4.53 ± 0.09	2.60 ± 0.05	1.28 ± 0.04	1.46 ± 0.03	γ-Ni
	b2	57.78 ± 2.01	22.43 ± 1.03	6.06 ± 0.05	2.64 ± 0.04	3.11 ± 0.11	3.31 ± 0.09	2.03 ± 0.07	2.64 ± 0.08	γ-Ni
	b3	60.89 ± 2.66	6.78 ± 0.16	3.32 ± 0.07	4.21 ± 0.10	1.03 ± 0.04	20.19 ± 0.66	2.22 ± 0.07	1.36 ± 0.05	TiNi_3_
	b4	14.40 ± 0.88	54.52 ± 1.96	23.15 ± 1.03	1.11 ± 0.02	1.10 ± 0.03	2.48 ± 0.03	1.52 ± 0.02	1.72 ± 0.05	(Cr, Mo)
Region C	c1	57.77 ± 1.69	13.66 ± 0.89	6.96 ± 0.36	2.71 ± 0.03	3.35 ± 0.06	12.64 ± 0.55	1.04 ± 0.05	1.87 ± 0.06	γ-Ni + TiNi_3_
	c2	3.11 ± 0.04	64.22 ± 2.34	24.83 ± 1.56	1.03 ± 0.02	2.29 ± 0.07	2.11 ± 0.03	1.13 ± 0.03	1.28 ± 0.06	(Cr, Mo)
Region D	d1	11.19 ± 0.66	6.28 ± 0.15	1.82 ± 0.04	1.60 ± 0.03	1.87 ± 0.09	68.82 ± 2.65	3.53 ± 0.08	4.89 ± 0.09	β-Ti
	d2	27.53 ± 1.56	1.32 ± 0.05	1.14 ± 0.04	1.04 ± 0.02	1.80 ± 0.05	60.49 ± 2.34	3.32 ± 0.06	3.36 ± 0.08	Ti_2_Ni
	d3	27.70 ± 1.33	2.83 ± 0.09	1.29 ± 0.05	1.19 ± 0.05	1.35 ± 0.04	62.73 ± 2.08	1.90 ± 0.06	1.01 ± 0.05	Ti_2_Ni
Region E	e1	1.56 ± 0.05	3.43 ± 0.09	1.63 ± 0.05	1.15 ± 0.05	1.40 ± 0.04	78.30 ± 3.01	9.39 ± 0.89	3.14 ± 0.04	α-Ti
	e2	5.08 ± 0.15	4.11 ± 0.11	1.64 ± 0.05	1.29 ± 0.04	1.45 ± 0.04	73.98 ± 3.21	5.94 ± 0.43	6.51 ± 0.28	β-Ti
	e3	5.37 ± 0.11	3.51 ± 0.09	1.72 ± 0.06	1.22 ± 0.03	1.56 ± 0.03	75.71 ± 2.99	4.41 ± 0.13	6.50 ± 0.38	β-Ti
	e4	29.45 ± 1.03	1.91 ± 0.09	1.33 ± 0.08	1.13 ± 0.07	1.06 ± 0.08	57.19 ± 1.87	6.36 ± 0.06	1.57 ± 0.04	Ti_2_Ni
	e5	28.51 ± 1.01	1.00 ± 0.03	1.05 ± 0.04	1.11 ± 0.06	1.42 ± 0.05	63.92 ± 1.88	1.47 ± 0.05	1.52 ± 0.03	Ti_2_Ni
	e6	26.90 ± 1.23	1.80 ± 0.10	1.12 ± 0.09	1.02 ± 0.03	1.28 ± 0.02	64.41 ± 2.13	1.92 ± 0.04	1.55 ± 0.05	Ti_2_Ni
Region F	f1	-	-	-	-	-	88.99 ± 2.44	8.76 ± 0.26	2.25 ± 0.04	α-Ti
	f2	-	-	-	-	-	86.73 ± 2.29	3.60 ± 0.05	9.67 ± 0.18	β-Ti

**Table 5:** Phase type and volume fraction of the transition regions of IN625/TC4 HS.

Region	Phase Type and Volume Fraction (%)						
	γ-Ni	laves	(Cr, Mo)	TiNi_3_	Ti_2_Ni	β-Ti	α-Ti
Region A	81.4	17.1	-	-	-	-	-
Region B	73.2	-	3.0	23.0	-	-	-
Region C	19.2	-	38.4	38.1	-	-	-
Region D	-	-	-	-	39.4	57.7	
Region E	-	-	-	-	35.1	51.3	9.3
Region F	-	-	-	-	-	8.7	90.6

## Data Availability

The original contributions presented in this study are included in the article. Further inquiries can be directed to the corresponding author.
